# SpeechMatch—A novel digital approach to supporting communication for neurodiverse groups

**DOI:** 10.1049/htl2.12090

**Published:** 2024-09-05

**Authors:** Sarah Lennard, Samuel J. Tromans, Robert Taub, Sarah Mitchell, Rohit Shankar

**Affiliations:** ^1^ Department of Intellectual Disability Neuropsychiatry, Research Team Cornwall Partnership NHS Foundation Trust Truro UK; ^2^ Cornwall Intellectual Disability Equitable Research (CIDER) University of Plymouth Peninsula School of Medicine Truro UK; ^3^ Department of Population Health Sciences, College of Life Sciences University of Leicester Leicester UK; ^4^ Adult Learning Disability Service Leicestershire Partnership NHS Trust Leicester UK; ^5^ The Arts Institute University of Plymouth Plymouth UK

**Keywords:** Autism, Autism spectrum disorder, speech, speech processing, technology

## Abstract

Communication can be a challenge for a significant minority of the population. Those with intellectual disability, autism, or Stroke survivors can encounter significant problems and stigma in their communication abilities leading to worse health and social outcomes. SpeechMatch (https://www.speechmatch.com/) is a digital App which is a pragmatic mobile language training platform that teaches individuals to “match” critical components of conversation and looks to provides subjects with immediate visual feedback to shape identification and expression of emotion in speech. While it has been used in autistic people there has been no systematic exploration of its strengths and weaknesses. Further, it's potential to afford improvements in communication to other vulnerable groups such as intellectual disability or Stroke survivors has not been explored. This study looked to understand acceptability from people with intellectual disability and/or autism and those recovering from a stroke on the utility and scope of SpeechMatch using co‐production techniques using experts by experience and a mixed methods evaluation. Results across four domains suggest high acceptability levels but highlighting needs for platform capabilities improvement and better user engagement. The study outlines a vital and essential aspect for improving SpeechMatch. It gives a template for evidenced based quality improvement of similar devices.

## 1 INTRODUCTION

Within the general population, communication skills are often taken for granted. Emotional reciprocity allows us to function in society. In everyday conversation, most people intuitively match several key parameters of speech namely volume, pacing, and emotional content. Language disorders can affect a wide range of people, including expressive, receptive, and mixed receptive‐expressive disorders. We set out to test a novel way of supporting and improving communication across groups of people with significant communication deficits using a digital tool, SpeechMatch (https://www.speechmatch.com/). The key groups focused on are described below‐

### 1.1 Autism spectrum disorder

Autism spectrum disorder (hereafter referred to as autism) is a neurodevelopmental condition characterised by deficits in social communication and social interaction, as well as a repertoire of ‘restricted, repetitive patterns of behaviour, interests, or activities’ [1]. Autism diagnostic criteria tend to focus on deficits/difficulties, but it is also important to recognise that many autistic people have special abilities related to their condition, including relating to memory, visuospatial skills, drawing, music, and computation [2].

Approximately 1% of the general population is autistic [3], with autism ranked as the 9th greatest cause of disability‐adjusted life years among all neurological conditions as of 2021 [4].

Autism is more prevalent in certain groups, including people with intellectual disability (ID) [5], and patients using mental health services [6, 7]. Many autistic people are undiagnosed, and groups with a greater likelihood of being undiagnosed include older people [8], women [9], and members of ethnic minority groups [10]. Compared to the general population, autistic people experience a significantly greater burden of mental illness [11] and physical illness [12], as well as a reduced life expectancy [13]. Furthermore, unemployment is very high among diagnosed autistic people, with the Office for National Statistics estimating that only 29.0% (95% confidence interval 24.8–33.2) of autistic people aged 16–64 years are in employment [14].

Speech is a core functional deficit in autistic people (in this article, we use identity‐first language when referring to autistic people, as this is in line with the preferences of the majority of the autistic community [15]), with them often struggling to match speech patterns in conversation [16]. Difficulties in communicating in socially expected ways can lead to loops of social avoidance and isolation. Support with social and emotional recognition is important to ensuring that autistic individuals can thrive in society.

### 1.2 Intellectual disability (ID)

Intellectual disability (ID, also known as learning disability and intellectual developmental disorder), refers to deficits in intellectual functioning (including in domains such as problem‐solving and abstract thinking) and deficits in adaptive functioning (including in domains such as social participation and occupational functioning), with onset during the developmental period [17]. ID has a global prevalence of around 1% [18], with idiopathic ID ranked as the 18th greatest cause of disability‐adjusted life years among all neurological conditions as of 2021 [4]. Communication difficulties are reported widely in the ID community, a national cross‐sectional study of 601 adults with ID [19], reporting that ‘57.9% of participants experienced communication difficulties, with 23.5% reporting severe difficulties’.

### 1.3 Stroke

A stroke is defined as ‘a serious life‐threatening medical condition that happens when the blood supply to part of the brain is cut off’ [20]. Strokes are highly prevalent both within the UK and globally, with stroke incidence in the UK predicted to increase by ‘60% per year between 2015 and 2035 [21]. As of 2021, stroke is ranked as the greatest cause of disability‐adjusted life years among all neurological conditions [4]. Following a stroke, language disorders are common, including ‘aphasia, alexia, agraphia and acalculia [22].

#### 1.3.1 SpeechMatch theory and impact

The literature in psychology and language points to “matching” or “vocal congruence” as the key to effective social interaction. Language studies also show abnormalities in autistic speech [23], and FMRI brain‐imaging studies show differences in language lateralization [24]. Studies have also revealed that autistic children and adults have marked difficulties in speech perception [25].

SpeechMatch (https://www.speechmatch.com/) is a digital therapeutic App for iPhone and iPad that was developed as a tool for shaping social conversational skills in autistic individuals [26] (https://www.myautism.org/news‐features/speechmatch‐match‐that‐pitch) and has been awarded a patent by the United States Patent and Trademark Office (USPTO).

Users receive immediate visual feedback regarding their progress in matching critical components of speech, specifically pitch (emotional content), rhythm (pacing), and volume. The goal is to improve the matching of these key components of speech regarding a range of emotions and expressions (happy, sad, pleasant surprise, unpleasant surprise, sarcastic, funny, and neutral statements).

Critically, SpeechMatch offers data‐driven real‐time metrics, assessments, and tracking of progress. SpeechMatch also offers personalisation and customisation features to enable subjects to use the app at any time with family and friends, yet also share data and progress with speech therapists if desired. All data is encrypted. There is developing evidence of significant progress made by autistic participants who use SpeechMatch in a consistent manner to improve their social language [27, 28].

SpeechMatch has been previously shown to be an effective digital tool that enables autistic people to shape their speech in ways that would enable them to participate more fully in societal social interactions [27]. However, there is a lack of structured evaluation of how, where and for whom in the communication support pathway SpeechMatch would be best suited.

### 1.4 Aim

The aim of this work is to understand acceptability from people with ID and/or autism and those recovering from a stroke on the utility and scope of SpeechMatch.

## 2 METHODS

A co‐production day in the UK was undertaken to explore the applicability of SpeechMatch to people with ID and/or autistic people, as well as people who had suffered a stroke and were faced with subsequent challenges of regaining speech. This stroke category had not previously been explored (although it had been considered), nor had the broader category of ID.

We used SpeechMatch on this co‐production day to examine if it:

- Can be used outside of official (and often limited) therapy sessions, that can be used in a family setting and/or with friends, and that can create a community of peer support.
- Is data‐driven and therefore can it help monitor progress.
- Is considered easy and fun to use and has features that can be personalised/customised for individual users and their families.
- Can be further developed with additional features, including elements of gamification.

We invited people with the relevant diagnosis/diagnoses to attend in two separate sessions. Their family members, carers or friends were also invited to attend. Each participant and his/her carer were given an iPad pre‐loaded with SpeechMatch. Only initial minimal guidance was provided by the app developer to enable each participant to play with the app, and to determine their own route and preferences in using the app. General guidance continued to be provided if requested throughout the 2‐h session. Toward the end of the session, a paper questionnaire about the general use of the app was distributed (Supporting Information).

### 2.1 Survey development

The survey questions were developed collaboratively by the authors. The Survey had six questions of which four were Likert style and two inviting free‐text opinions. The Likert questions captured the respondent's ease of use of SpeechMatch, helpfulness of the App, day‐to‐day utility and perceived self‐benefit on communication. A descriptive analysis was undertaken of the responses.

The free text responses inquired of two questions:

- Question 1: ‘Are there any changes to the app that you would recommend?’
- Question 2: ‘Are there any features of SpeechMatch that you particularly like?’

We also asked our stroke group an additional question, ‘do you think digital therapies, like SpeechMatch, might be useful tools in addition to person‐to‐person sessions with therapists?’. These qualitative results were analysed and grouped into themes for each question.

### 2.2 Ethics and governance

The NHS Health research authority tool (http://www.hra‐decisiontools.org.uk/research/index.html) confirmed no formal NHS Ethics approval was required (Supporting Information). All participants were advised at the start of the study that participation was voluntary and their replies if they chose to participate, would be anonymised and analysed. Written informed consent was taken from all participants. No participant identifier data was collected. Data was pooled prior to analysis. Further, it was to a participant group who were participating in co‐production as experts by experience.

## 3 RESULTS

The morning session included five participants with ID and/or autism. The afternoon session had two stroke patients and one person with epilepsy and communication issues.

In addition, three others who were supporting a participating member of one of the groups also participated in the feedback.

**FIGURE 1:**
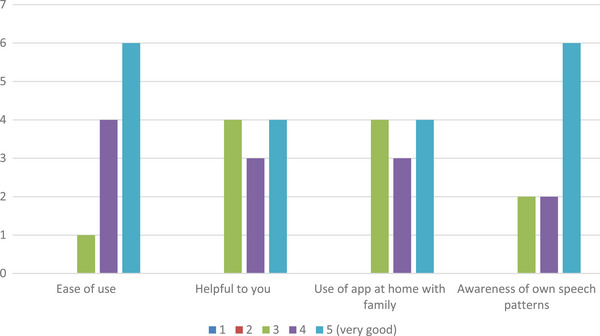
Survey results.

Figure 1 shows the results of the four Likert questions. The results from those with the relevant diagnosis and their carers have been combined. Of the eleven respondents, six found 100% satisfaction with ‘ease of use’, four 80%, and one 60%. Four people each for 100% and 60%, respectively, found SpeechMatch as being ‘personally helpful’ while three suggested it to be 80%, this was the same feedback for the ‘day‐to‐day utility’ question. When it came to awareness of their own speech pattern, six found 100% satisfaction while two each told of 60% and 80% satisfaction with one having no response.

In response to the open question one, thematic analysis of the group comments was focused on the interface, improving the looks and colours of the app with the second theme wanting a visual tutorial and feedback without the use of percentages as feedback. The other theme that was across much of the group for this question was the need to slowdown the audio to be able to understand what was being said to allow for people with intellectual disabilities and/or autism to process the content.

In response to question two, themes generated from the responses were the need to improve tone and pitch. A further theme on the scoring system was commented on across the groups of a way of competing against yourself to improve, with one comment ‘I like that you can work on your score’.

The additional question asked of the stroke group generated again the theme generated of interface issues and personalisation. With one participant commenting ‘Can offer privacy when not confident enough to practise in front of other people.’

## 4 DISCUSSION

The aim of the focus group was to understand the acceptability of the SpeechMatch app to a UK‐based group of people with communication disorders including some with ID and/or autism and a second group who have had a stroke. Further development of SpeechMatch has been informed by the co‐production group. The synthesis of the discussion suggests SpeechMatch would benefit from:

- Incorporation of additional features that enhance the platform's capabilities and enhance the overall user experience.
- Building a comprehensive plan for use.
- Ensuring that both points above are further reviewed by experts with appropriate experience.

## 5 LIMITATIONS

This was a small group, however, this allowed the investigation team to introduce the app giving each person ample time to play with the app.

In the case of autistic people as was found in the variability of our feedback it is worth noting that some may not want to change their conversational habits and may take issue with SpeechMatch if it were presented to ‘correct’ their speech differences. This stance is related to the double empathy problem theory described by Milton [29]. Historically, the ‘blame’ for difficulties in social interaction and communication between autistic and non‐autistic people has been principally laid at the feet of the autistic community, whose communication style has been viewed as abnormal relative to their non‐autistic peers, rather than both communication styles being recognised as valid. This theory has been supported by subsequent research that often the mismatch in communication styles between autistic and non‐autistic people causes difficulties [30].

Thus, SpeechMatch should be considered for people who are motivated to change their communication style, with their personal choices respected, rather than presented as a treatment imposed on a community such as autism at large.

## 6 CONCLUSION

By understanding the acceptability of the tool (SpeechMatch) and understanding feedback from those who used SpeechMatch on the co‐production day, the learning was to broaden and incorporate additional features in SpeechMatch that would enable it to be helpful to a broader group of patients. Furthermore, the study gives a template for evidenced‐based quality improvement of similar devices.

## AUTHOR CONTRIBUTIONS

**Sarah Lennard**: Conceptualization; data curation; formal analysis; investigation; methodology; validation; visualization; writing—original draft. **Samuel J. Tromans**: Conceptualization; investigation; methodology; visualization; writing—review and editing. **Robert Taub**: Conceptualization; formal analysis; funding acquisition; investigation; project administration; supervision; validation; visualization; writing—review and editing. **Sarah Mitchell**: Conceptualization; investigation; methodology; visualization; writing—review and editing. **Rohit Shankar**: Conceptualization; data curation; formal analysis; funding acquisition; investigation; methodology; project administration; resources; supervision; validation; visualization; writing—review and editing.

## CONFLICT OF INTEREST STATEMENT

Robert Taub is the developer and inventor of Speech‐Match, the product discussed significantly in the paper. Rohit Shankar has received institutional and research support from LivaNova, UCB, Eisai, Veriton Pharma, Neuraxpharm, Bial, Angelini, UnEEG, and Jazz/GW pharma outside the submitted work. No other author has any declared conflict of interest related to this paper.

## Supporting information

Supporting Information 1

Supporting Information 2

## Data Availability

All data used for the paper is within the manuscript.
