# Supplementary material for: SpeechMatch—A novel digital approach to supporting communication for neurodiverse groups
Source: Healthc Technol Lett. 2024 Sep 5;11(6):447–51. doi: 10.1049/htl2.12090 (PMC11665792; doi:10.1049/htl2.12090)
Supplement: Supplementary file 1 — Supporting Information 1 [file HTL2-11-447-s001.docx]

Supplementary information one: Survey Questionnaires templates

| **Feedback form Morning Session** | |  | **1** | **2** | **3** | **4** | **5** |  |
| --- | --- | --- | --- | --- | --- | --- | --- | --- |
| 1 | Did you find the SpeechMatch App easy to use? | Not easy |  |  |  |  |  | Very easy |
| 2 | Do you think using this App could be helpful to you? | Not helpful |  |  |  |  |  | Very helpful |
| 3 | Would you use the App at home with family or friends? | Not really |  |  |  |  |  | Many times |
| 4 | Did using SpeechMatch make you more aware of your own speech patterns? | Not really |  |  |  |  |  | Very aware |
|  | | | | | | | | |
| 5 | Are there any changes to the App that you would recommend? | | | | | | | |
| 6 | Are there any features of SpeechMatch that you particularly like? | | | | | | | |

| **Feedback Form for family members in morning session** | |  | **1** | **2** | **3** | **4** | **5** |  |
| --- | --- | --- | --- | --- | --- | --- | --- | --- |
| 1 | Would you use SpeechMatch within your family? | Not really |  |  |  |  |  | Many times |
| 2 | Did you find the “custom phrase” feature helpful? | Not helpful |  |  |  |  |  | Very helpful |
|  | | | | | | | | |
| 3 | Do you have suggestions for improving SpeechMatch? | | | | | | | |
| 4 | Would you like to participate in additional studies with SpeechMatch? | | | | | | | |
| 5 | Do you think SpeechMatch could improve attention to social cues? | | | | | | | |

| **Feedback Form Afternoon Session** | |  | **1** | **2** | **3** | **4** | **5** |  |
| --- | --- | --- | --- | --- | --- | --- | --- | --- |
| 1 | Did you find the SpeechMatch App easy to use? | Not easy |  |  |  |  |  | Very easy |
| 2 | Do you think using this App could be helpful to you? | Not helpful |  |  |  |  |  | Very helpful |
| 3 | Would you use the App at home with family or friends? | Not really |  |  |  |  |  | Many times |
| 4 | Did using SpeechMatch make you more aware of your own speech patterns? | Not really |  |  |  |  |  | Very aware |
|  | | | | | | | | |
| 5 | Are there any changes to the App that you would recommend? | | | | | | | |
| 6 | Are there any features of SpeechMatch that you particularly like? | | | | | | | |

| 7 | Following a neurological event such as a stroke, people end up in hospital or a rehab centre. A goal is to maximise therapy time, even when therapists (e.g. speech or occupational) are not available.  Do you think digital therapies, like SpeechMatch, might be useful tools in addition to person to person sessions with therapists? |
| --- | --- |
